# Retrograde inferior vena caval perfusion for total aortic arch replacement surgery: a randomized pilot study

**DOI:** 10.1186/s12872-021-02002-9

**Published:** 2021-04-20

**Authors:** Jing Lin, Zhen Qin, Xinhao Liu, Jiyue Xiong, Zhong Wu, Yingqiang Guo, Deying Kang, Lei Du

**Affiliations:** 1 Department of Anesthesiology, West China Hospital, Sichuan University, No. 37 Guo Xue Alley, Chengdu, 610041 Sichuan China; 2 Department of Cardiovascular Surgery, West China Hospital, Sichuan University, No. 37, Guo Xue Alley, Chengdu, 610041 Sichuan China; 3 Department of Evidence-Based Medicine and Clinical Epidemiology, West China Hospital, Sichuan University, No. 37, Guo Xue Alley, Chengdu, 610041 Sichuan China

**Keywords:** Retrograde inferior vena caval perfusion, Antegrade cerebral perfusion, Total aortic arch replacement surgery

## Abstract

**Objectives:**

Antegrade cerebral perfusion (ACP) under moderate hypothermic circulatory arrest is used during total aortic arch replacement surgery (TARS) in patients with acute type A aortic dissection, but it is associated with high mortality and morbidity. We hypothesized that combining ACP with retrograde inferior vena caval perfusion (RIVP) improves outcomes.

**Methods:**

This pilot study was prospective, randomized, controlled and assessor-blinded. Patients scheduled for TARS were randomly treated with either ACP or RIVP + ACP. The primary outcome was a composite of mortality and major complications including paraplegia, postoperative renal failure, severe liver dysfunction, and gastrointestinal complications. Secondary outcomes included neurological complications, length of intubation and requirement of blood products.

**Results:**

A total of 76 patients were recruited (n = 38 per group). Primary outcome occurred in 23 patients (61%) in the ACP group and 16 (42%) in the RIVP + ACP group (OR: 0.60, 95% CI: 0.21–1.62; *p* = 0.31). There was a lower incidence of transient neurological deficits in the RIVP + ACP group (26% vs. 58%, OR: 0.26; 95% CI: 0.10–0.67,*p* = 0.006;). The RIVP + ACP group underwent shorter intubation (25 vs 47 h, *p* = 0.022) and required fewer blood products (red cells, 3.8 units vs 6.5 units, *p* = 0.047; platelet: 2.0 units vs 2.0 units, p = 0.023) compared with the ACP group.

**Conclusions:**

RIVP + ACP may be associated with lower incidence of transient neurological deficits, shorter intubation and less blood transfusion requirement than ACP alone during TARS. Multi-center, randomized trials with larger samples are required to determine whether RIVP + ACP is associated with lower rates of mortality and major complications.

*Trial registration*: Pilot study of a RCT registered in clinicaltrials.gov (NCT03607786), Registered 30 July, 2018—Retrospectively registered, https://clinicaltrials.gov/ct2/show/NCT03607786.

**Supplementary Information:**

The online version contains supplementary material available at 10.1186/s12872-021-02002-9.

## Introduction

Acute type A aortic dissection (AAAD) is a life-threatening condition [1–3] that, if it involves the aortic arch and distal aorta, usually requires immediate surgery to replace both the ascending aorta and aortic arch with artificial vascular grafts in a procedure known as total aortic arch replacement surgery (TARS). Because TARS requires open the distal anastomosis of the graft and descending aorta, patients may undergo a period of circulatory arrest. To minimize ischemic injury, Griepp et al*.* recommended in 1975 that patients be put under deep hypothermic circulatory arrest (DHCA) [4]. However, DHCA is associated with high postoperative mortality (13–40%) [5, 6] and morbidity [7–9]. Modern techniques such as selective antegrade or retrograde cerebral perfusion (ACP or RCP) under moderate hypothermic circulatory arrest (MHCA) have substantially reduced these risks and are now widely used all over the world [10–13]. Nevertheless, 30-day mortality can still be as high as 5.3–19% [14, 15]; stroke incidence, 6.7–10% [16, 17]; and acute kidney injury, 19–54% [18, 19].

Reducing ischemic injury to the viscera and lower body is key to further improve outcomes in patients undergoing TARS. Recently, we reported that retrograde inferior vena caval perfusion (RIVP) while opening the distal anastomosis may help provide the lower body with oxygenated blood during circulatory arrest [20]. Here, we report the results of a pilot study in which we compared postoperative outcomes in patients undergoing ACP or RIVP + ACP during TARS. The primary purpose of this small trial was to initially explore the safety and outcomes of RIVP to guide a large prospective trial.

## Materials and methods

This was a prospective, randomized, controlled, and assessor-blinded study. Patients older than 18 years were included if they had been diagnosed with AAAD based on computed tomography angiography and scheduled for emergency TARS by two surgeons (YQG and ZW) in the Cardiovascular Department of West China Hospital of Sichuan University between April 1 and December 31, 2018. Patients were excluded if they were pregnant, unable to understand or give informed consent, or were already participating in another clinical trial that might interfere with the outcomes of our trial. Exit criteria included hemi-arch replacement, retrograde cerebral perfusion, or withdrawal of consent. TARS was performed when the intimal flap appeared in the ascending aorta, aortic arch and distal aorta. Hemiarch replacement was performed if the intimal flap was restricted in the ascending aorta and the aortic arch had a diameter within the normal range without distal malperfusion [21].

This is a pilot study of a large, prospective, assessor-blinded clinical trial that has been registered at clinicaltrial.gov (ID: NCT03607786) and approved by the Medical Ethics Committee of West China Hospital, Sichuan University (No. 201824). The enrolled patients in this pilot study would not be included into the on-going large clinical trial. The protocol followed the guidelines of the Declaration of Helsinki. Written informed consent was obtained from all patients and their relatives preoperatively, and they were informed that they could leave the study at any time for any reason, without consequences.

### Anesthesia, surgery and cardiopulmonary bypass

Patients were managed as described [22]. Briefly, general anesthesia was induced and maintained by sevoflurane inhalation and intravenous infusion of propofol, sufentanil and cisatracurium. Invasive blood pressure in the bilateral radial arteries and left dorsalis pedis artery as well as nasopharyngeal and rectal temperature were regularly monitored. A central venous catheter was inserted through the right internal jugular vein. Cerebral perfusion was monitored using near-infrared spectroscopy (NIRS; EGOS-600A, Suzhou Engine Bio-medical Electronics, Suzhou, China). A transesophageal echocardiographic probe was routinely placed.

After median sternotomy, arterial cannulation was performed in the distal ascending aorta or aortic arch or in the right axillary, innominate, or femoral arteries depending on the dissection condition and the surgeon's preference. The superior and inferior vena cava were cannulated for venous drainage. Cardiopulmonary bypass (CPB) was performed using two rolling pumps, membrane oxygenator, and tubes. To prepare for RIVP, the arterial line was bifurcated after pump 1 and the oxygenator. One branch of the arterial line was designed for systemic perfusion, which allowed ACP during circulatory arrest. The other branch was connected to pump 2, a pressure monitor, and a drainage tube from the inferior vena cava, which allowed RIVP during circulatory arrest (Video). During RIVP, the circuit pressure after the RIVP pump was monitored to assess the pressure in the inferior vena cava.

Systemic cooling was initiated immediately after the start of CPB, and the patient’s head was packed in ice during cooling. After the ascending aorta was cross-clamped, myocardial protection was achieved by infusion of cold blood cardioplegia delivered intermittently in antegrade or retrograde mode. Proximal aortic root repair was performed during cooling. The Bentall procedure was performed if patients had severe aortic regurgitation and dilation of the aortic root.

TARS was performed using the elephant-trunk technique and a four-branched graft [23]. Circulatory arrest was initiated after the target temperature was reached. The supra-aortic vessels were clamped while the ascending aortic clamp and aortic cannula were removed upon initiation of ACP. The arch was resected distally to the opening of the left subclavian artery, with each head vessel prepared for individual anastomosis. A stent endograft (CRONUS, MicroPort Scientific Corporation, Shanghai, China) was inserted into the true lumen of the descending aorta in an antegrade manner and the aortic wall was anastomosed to the graft end. Systemic circulation was resumed through a side branch of the arch graft to perfuse the lower body. The arch reconstruction was performed by anastomosis of the left common carotid artery, left subclavian artery and innominate artery with the other three branches of the graft. Patients were progressively weaned from CPB.

Blood components were infused to maintain post-CPB hematocrit and coagulopathy. All patients were transferred back to the intensive care unit after the operation.

### Study groups

After arrival in the operating room, patients were randomly assigned to the ACP or RIVP + ACP group by an independent coordinator who used a computer-generated randomization list. Outcome assessors were blinded to patient allocation, while the surgeon, anesthesiologist, perfusionist and operating room personnel were not.

For patients in the control group, ACP group, ACP was initiated during circulatory arrest by either axillary or direct innominate artery cannulation using a 10- to 14-Fr perfusion cannula at a rate of 5–12 mL/kg/min under a pump pressure of 50–70 mmHg. Bilateral ACP was employed when the NIRS value decreased for more than 20% of baseline.

For patients in the RIVP + ACP group, RIVP was initiated after the inferior vena cava cannula was snared and the distal end of the inferior vena cava drainage tube was cross-clamped. RIVP was performed using pump 2 and oxygenated blood from the arterial line; flow was slowly increased to 5–10 mL/min/kg to achieve a target circuit pressure of 20–25 mmHg (Video). After that, the aorta was opened for stent and anastomosis. RIVP was discontinued after anastomosis of the descending aorta, and the graft was completed. ACP and all other steps were performed as for patients in the ACP group.

### Outcomes

To determine the effect of RIVP on patient outcomes and lower body ischemia in particular, we defined the primary outcome as a composite of organ dysfunction in the lower body (including paraplegia, postoperative dialysis-dependent renal failure, severe liver dysfunction, and gastrointestinal complications), as well as all-cause mortality, which occurred during hospitalization (regardless of length of stay) and for up to 30 days after surgery if the patient was discharged. Severe liver dysfunction was defined as a serum alanine level 8 times the upper normal level or a serum alanine level 3 times the upper normal level, as well as a total bilirubin level greater than 56 µmol/L. Gastrointestinal complications [24] included gastrointestinal bleeding, perforation, intestinal ischemia, pancreatitis, acute cholecystitis, and paralytic ileus. Secondary outcomes included stroke/cerebrovascular events, prolonged ventilation (> 48 h), transient neurological deficits (TND), myocardial infarction, acute kidney injury not requiring dialysis, surgical re-exploration for bleeding, deep sternal wound infection, length of intubation, length of hospital stay, blood product requirement, postoperative drainage and hemoglobin level. All patients with new central nervous system symptoms were assessed by a neurologist. Demographics and outcomes were defined as recommended by the Society of Thoracic Surgeons (www.sts.org/quality-safety/performance-measures). The details of definitions of the outcomes were as previously reported [22].

Clinical data from the initial hospitalization were prospectively entered into our institutional database. Discharged patients were assessed directly in our outpatient clinic or by telephone. Follow-up was completed for all patients by February 1, 2019.

### Statistical analysis

Data were analyzed using SPSS 22.0 (IBM, Armonk, NY, USA). Differences associated with *p* < 0.05 were considered statistically significant. Normality of the distribution of continuous data was assessed using the *Shapiro-Wilks* test, for which *P* > 0.05 was taken to indicate normal distribution. Variables showing a normal distribution were expressed as mean ± standard deviation, and differences were assessed for significance using an independent t test. Other variables were expressed as median (interquartile range), and differences were assessed using the *Mann–Whitney U* test. Categorical variables were expressed as numbers (percentages), and differences were assessed using a *Chi-Squared* test.

Binary logistic regression was used to calculate odds ratios (ORs) among. Primary outcome was adjusted for age, gender, body mass index (BMI). Transient neurological deficits were adjusted for age, gender, BMI, lowest nasopharyngeal temperature, unilateral/bilateral cerebral perfusion, and cerebral flow. Prolonged intubation was adjusted for age, gender, BMI, and volume of red cell transfusion.

We used generalized estimating equations to assess the significance of inter-group differences in chest drainage volume and hemoglobin level. We used robust standard errors and the working correlation structure specified as autoregressive.

## Results

### Participants

**Fig. 1:**
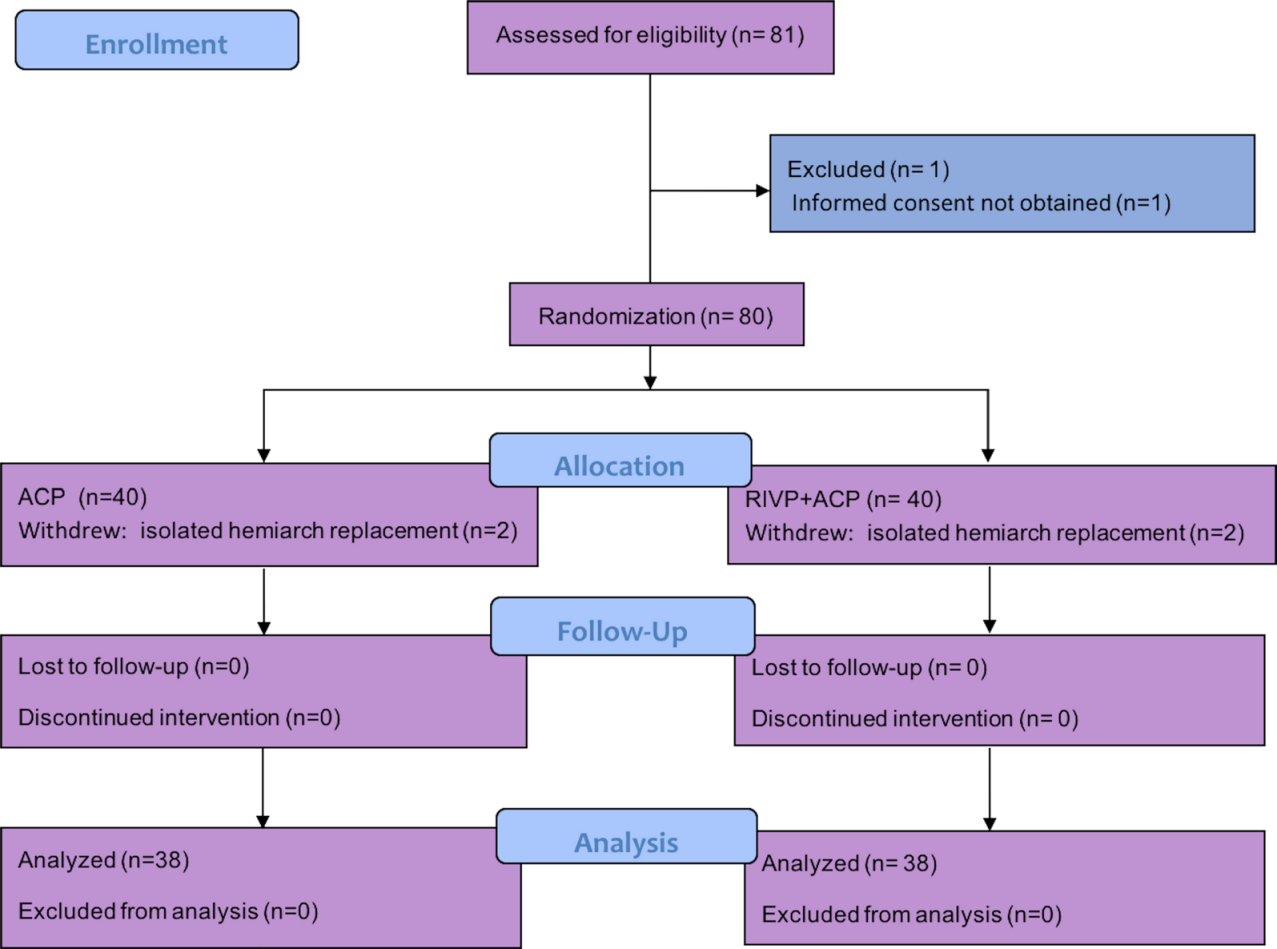
Flowchart of the study population. A total of 76 patients were enrolled in the final analysis based on the inclusion and exclusion criteria. ACP: antegrade cerebral perfusion; RIVP: retrograde inferior vena caval perfusion

A total of 81 patients scheduled for TARS were screened. One was excluded because informed consent could not be obtained, leaving 80 patients who entered into the study and were randomized. Of these, 4 withdrew because hemi-arch replacement was needed (n = 2 in each group), leaving a total of 76 patients (mean age: 45 ± 10 years, 65 males) with 38 patients per group (Fig. 1). Patient baseline characteristics, including demographic characteristics, comorbidities, laboratory indices and preoperative treatments, were similar between groups. An exception was C-reactive protein level, which was slightly higher in the RIVP + ACP group than in the ACP group (40.8 vs 77.5 mg/L, *p* = 0.05) (Table 1).

**Table 1:** Baseline variables in patients treated with ACP or RIVP + ACP during TARS

Variable	ACP group (n = 38)	RIVP + ACP group (n = 38)	*P value*
**Demographics**			
Age	45 ± 10	47 ± 10	0.49
Male, n (%)	30 (79)	35 (92)	0.10
Ethnicity, n (%)			1.00
Han ^a^	36 (95)	37 (97)	
Tibetan ^a^	1 (3)	1 (3)	
Other ^a^	1 (3)	0 (0.0)	
Body mass index, kg/m^2^	24.64 ± 3.71	25.51 ± 3.20	0.41
Current smoker, n (%)	20 (53)	18 (47)	0.65
Current drinker, n (%)	5 (13)	6 (16)	0.74
EuroScore II, median (IQR)	2.49 (2.38, 4.12)	2.68 (1.98, 3.86)	0.98
**Circulatory system**			
Hyperlipemia, n (%)^a^	1 (3)	1 (2.6)	1.00
Hypertension, n (%)	27 (71)	21 (55)	0.15
Ejection fraction, %	60 ± 9	60 ± 8	0.79
Arrhythmia, n (%)^a^	0 (0)	1 (3)	1.00
Cardiovascular intervention, n (%)	4 (11)	2 (5)	0.67
**Respiratory system**			
Respiratory failure, n (%)^a^	1 (3)	0 (0.0)	1.00
Infection, n (%)^a^	1 (3)	0 (0.0)	1.00
Atelectasis, n (%)^a^	1 (3)	2 (5)	1.00
Pleural effusion, n (%)	6 (16)	8 (21)	0.55
**Nervous system, n (%)**			0.12
Transient ischemic attack ^a^	0 (0.0)	1 (3)	
Stroke history ^a^	0 (0.0)	3 (8)	
Renal failure (RIFLE III) ^a^	0 (0.0)	0 (0)	-
Diabetes, n (%)^a^	1 (3)	0 (0.0)	1.00
Hypothyroidism, n (%)^a^	4 (11)	5 (13)	1.00
Liver dysfunction, n (%)^a^	1 (3)	2 (5)	1.00
Gastrointestinal bleeding, n (%)^a^	1 (3)	1 (3)	1.00
Ulcer, n (%)^a^	2 (5)	0 (0)	0.47
**Laboratory index**			
Hemoglobin (g/L)	134 ± 17	132 ± 18	0.61
Platelets (× 10^9^/L)	162 ± 72	187 ± 96	0.20
Leukocytes (× 10^9^/L)	10.30 ± 3.23	10.72 ± 3.56	0.59
International normalized ratio	1.02 (0.97, 1.12)	1.02 (0.97, 1.10)	0.69
Fibrinogen (g/dL)	3.45 ± 1.60	3.96 ± 1.75	0.19
Glucose (mmol/L)	6.14 ± 1.09	6.46 ± 1.02	0.19
C-reactive protein (mg/L)	40.8 (11.5, 86.0)	77.5 (36.3, 101.2)	0.05
Troponin-T (ng/L)	16.1 (9.9, 43.9)	20.1 (12.8, 34.2)	0.40
Creatinine level (umol/L)			0.45
≤ 110	28(74)	30(79)	
110–176	9(24)	8(21)	
≥ 176	1(3)	0(0)	
**Medicine, n (%)**			
Beta blocker	33 (87)	32 (84)	0.74
Aspirin	1(3)	1 (3)	1.00
Anticoagulants	1 (3)	0 (0)	1.00

Continuous data are expressed as mean ± SD or median (interquartile range); categorical data, as n (%)

*ACP* antegrade cerebral perfusion, *RIFLE III* risk, injury, failure, loss, and end-stage criteria, *RIVP* retrograde inferior vena caval perfusion, *TARS* total aortic arch replacement surgery

^a^Chi-Squared test

The RIVP + ACP group contained higher proportions of patients with aortic dissection involving celiac trunk artery (18% vs 45%, *p* = 0.014) and right renal artery (18% vs 40%, *p* = 0.043). The groups showed no significant differences in rates of involvement of other arteries (Table 2).

**Table 2:** Arteries involved in aortic dissection, expressed as n (%)

Artery	ACP group (n = 38)	RIVP + ACP group (n = 38)	*P value*
Coronary	12 (32)	5 (13)	0.054
Innominate	24 (63)	30 (79)	0.13
Left common carotid	18 (47)	20 (53)	0.65
Left subclavian	17 (45)	21 (55)	0.36
Celiac trunk	7 (18)	17 (45)	0.014
Left renal	13 (34)	16 (42)	0.48
Right renal	7 (18)	15 (40)	0.043

### Surgical characteristics

Times from first symptom to surgery and from admission to surgery were similar between the groups (Table 3). The two groups were also similar in the numbers of patients undergoing elephant trunk implantation, aortic valve replacement or coronary artery bypass grafting. During lower body circulatory arrest, the nasopharyngeal and rectal temperatures were 2.5 °C higher in the RIVP + ACP group than in the ACP group (Table 3). During circulatory arrest, ACP blood flow was lower, but the rate of bilateral ACP was higher, in the ACP group than in the RIVP + ACP group. Patients in the RIVP + ACP group had a blood flow of 11 ± 2 mL/min/kg for RIVP.

**Table 3:** Surgical characteristics in patients treated with ACP or RIVP + ACP during TARS

Variable	ACP group (n = 38)	RIVP + ACP group (n = 38)	*P value*
Time from first symptom to surgery (h)	66 (60, 130)	42 (29, 126)	0.26
Time from admission to surgery (h)	41 (22, 56)	26 (18, 36)	0.42
**Surgery type, n (%)**			
Elephant trunk	34 (90)	35 (92)	1.00
Aortic valve replacement	19 (50)	14 (37)	0.25
Aortic valvuloplasty ^a^	1 (3)	0 (0)	1.00
Coronary artery bypass grafting ^a^	2 (5)	1 (3)	1.00
**Arterial cannulation site, n (%)**^a^			0.80
Femoral artery	25 (66)	27 (71)	
Descending aorta	12 (32)	11 (29)	
Subclavian artery	1 (3)	0 (0)	
**Variables during lower body arrest**			
Lowest temperature (°C)			
Nasopharyngeal	25.3 (24.5, 25.7)	27.8 (27.6, 28.1)	< 0.001
Rectal	27.2 (25.9, 27.8)	28.9 (28.6, 29.3)	< 0.001
Antegrade cerebral perfusion			
Flow (mL/min/kg)	5.8 ± 1.3	7.2 ± 1.5	0.001
Unilateral/bilateral	14/24	31/7	< 0.001
Left radial artery pressure (mmHg)	15 ± 8	14 ± 7	0.46
Retrograde inferior vena caval perfusion			
Flow (mL/min/kg)	–	11 ± 2	–
Pressure (mmHg)	–	22 ± 3	–
Cardiopulmonary bypass times (min)			
From initiation to low body arrest	112 ± 33	120 ± 33	0.27
The specific circulatory arrest	34.4 ± 9.2	34.5 ± 8.3	0.96
From rewarming to weaning pump	151 ± 60	97 ± 23	< 0.001
Cross-clamping time	198 ± 55	189 ± 36	0.39
Surgery duration (hour)	8.27 (6.75,9.75)	8.08 (7.08,9.00)	0.33

Continuous data are expressed as mean ± SD or median (interquartile range); categorical data, as n (%)

*ACP* antegrade cerebral perfusion, *RIVP* retrograde inferior vena caval perfusion, *TARS* total aortic replacement surgery

^a^Chi-squared test

Time from CPB initiation to rewarming and aortic cross-clamping time were similar between groups, but the time from rewarming to weaning off the pump was shorter in the RIVP + ACP group (Table 3).

### Clinical outcomes

**Fig. 2:**
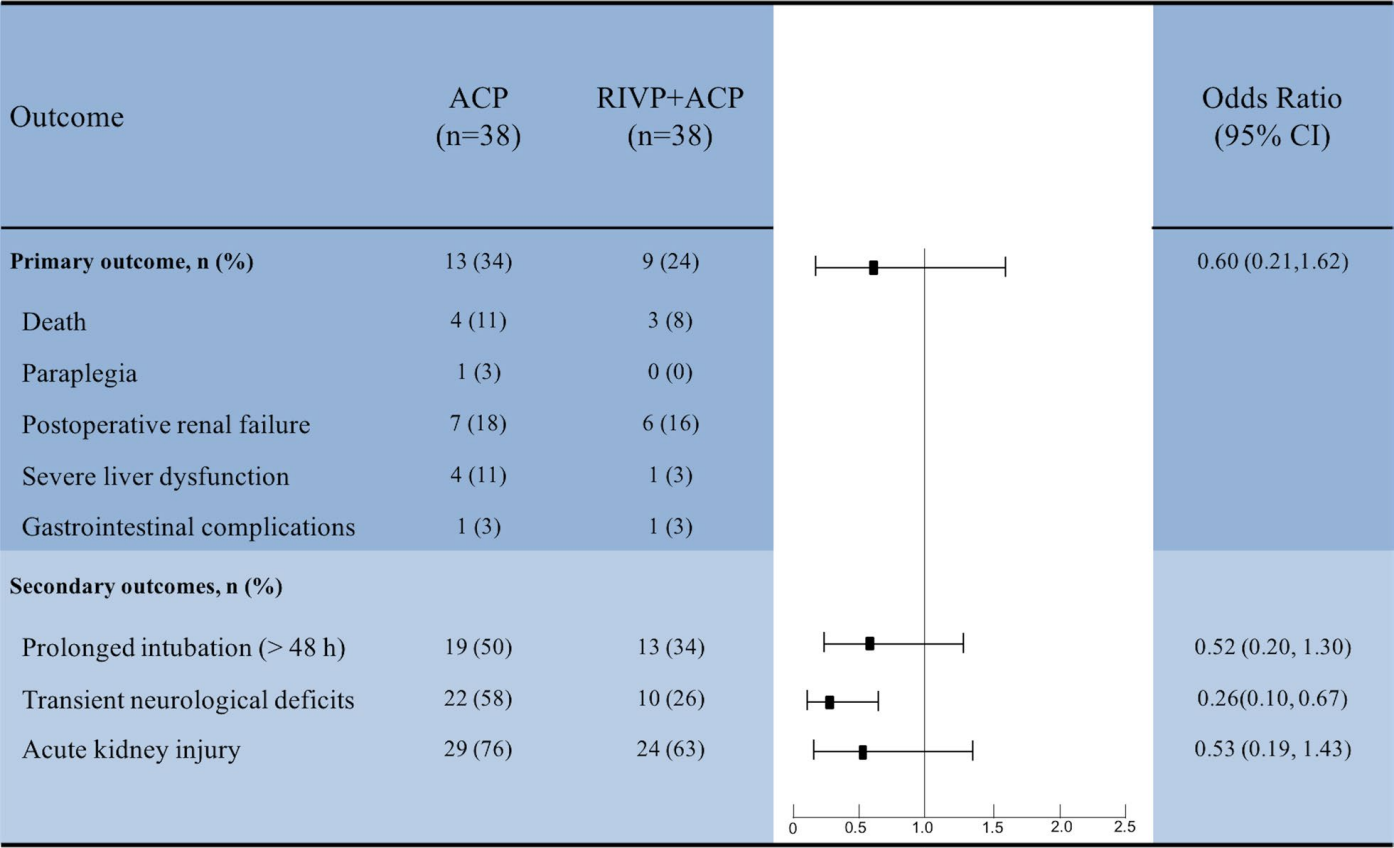
RIVP + ACP might be associated with reduced TND and less blood products transfusion. ACP, antegrade cerebral perfusion; RIVP, retrograde inferior vena caval perfusion; TND, transient neurological deficits

Primary outcome occurred in a total of 22 patients (29%), comprising 7 deaths (9%), 1 case of paraplegia (1%), 13 cases of postoperative renal failure (17%), 5 cases of severe liver dysfunction (7%), and 2 cases of gastrointestinal complications (3%). Of 22 patients with primary events, 13 (34%) were in the ACP group, and 9 (24%) in the RIVP + ACP group (Fig. 2). Of the 4 deaths in the ACP group, two were caused by cardiogenic shock, one was caused by stroke, and one was caused by rupture of the abdominal aortic dissection. Of the 3 deaths in the RIVP + ACP group, two were caused by stroke induced by right common carotid artery dissection, and one was caused by multiple organ dysfunction secondary to lung infection. Kidney function based on the RIFLE classification was similar between the two groups (Additional file 2: Table 1).

Based on binary logistic regression, crude and adjusted ORs of primary outcomes in patients treated with RIVP + ACP were 0.60 (95% CI: 0.21–1.62) and 0.97 (95% CI: 0.24–3.97) relative to patients treated with ACP (Fig. 2).

Stroke incidence was similar between the two groups. However, the incidence of TND was lower in the RIVP + ACP group. Based on logistic regression, crude and adjusted ORs of TND in patients treated with RIVP + ACP were 0.26(95% CI: 0.10–0.67) and 0.13 (95% CI: 0.03–0.51) relative to patients treated with ACP (Fig. 2).

Duration of intubation was shorter in the RIVP + ACP group than in the ACP group (25 h vs 47 h, p = 0.022). Crude and adjusted ORs of prolonged intubation in the RIVP + ACP group were 0.52 (95% CI: 0.20–1.30) and 0.56 (95% CI: 0.15–2.07) relative to ACP alone.

Length of hospital stays and rates of surgical re-exploration for bleeding and deep sternal wound infection were similar between the groups. The RIVP + ACP group showed a less requirement of blood products (RBC, 6.5 units vs 3.8 units, *p* = 0.047; PLT, 2.0 units vs 2.0 units, *p* = 0.023, Table 4) and higher hemoglobin level, but lower post-operative chest drainage volume (Additional file 2: Table 2).

**Table 4:** Other secondary adverse events in patients treated with ACP or RIVP + ACP during TARS

Outcome	ACP group (n = 38)	RIVP + ACP group (n = 38)	*P value*
Surgical re-exploration for bleeding	2 (5.3)	0 (0)	–
Deep sternal wound infection^a^	1 (2.6)	1 (2.6)	1.00
Length of intubation, hour^b^	47 (19, 89)	25 (13, 66)	0.022
Length of hospital stay, days^b^	16 (12, 20)	13 (10, 18)	0.36
Red blood cells, units^b^	6.5 (2.0, 13.0)	3.8 (1.5, 6.5)	0.047
Fresh frozen plasma, units^b^	2.0 (0, 4.7)	0.8 (0, 3.0)	0.26
Platelet, units^b^	2.0 (2.0, 3.0)	2.0 (2.0, 2.0)	0.023

Continuous data are expressed as median (interquartile range); categorical data, as n (%)

*ACP* antegrade cerebral perfusion, *RIVP* retrograde inferior vena caval perfusion, *TARS* total aortic replacement surgery

^a^Chi-squared test

^b^Mann–Whitney U test

## Discussion

RIVP with ACP is easy to be performed during TARS (Video). In this study, patients undergoing RIVP + ACP experienced lower incidence of transient neurological deficits, shorter intubation time and less requirement of blood products than those undergoing ACP alone, although the two groups did not differ significantly in the rate of composite events. These results suggest that RIVP combined with ACP might lead to better outcomes than ACP alone during TARS.

In this pilot study, RIVP at 22 ± 3 mmHg provided 11 ± 2 mL/min/kg of oxygenated blood to the lower body, and was associated with a 10% decrease in the rate of primary outcome. This reflects the fact that the RIVP + ACP group had a slightly lower RIFLE classification, although aortic dissection in that group more often involved the celiac trunk artery and right renal artery than in the ACP group. The protective effects may be explained by RIVP blood for providing continuous oxygenation to the lower body, as well as cooling and flushing out the particular embolic debris, similarly to retrograde superior vena caval perfusion [25].

In the present study, we increased body temperature in the RIVP + ACP group during circulatory arrest. We maintained patients at a nasopharyngeal temperature of 26–28 °C and a rectal temperature of 28–30 °C, which have been reported safe for RIVP patients [20]. Since the lower body is perfused through RIVP during the period of circulatory arrest, we believe that it is safe to elevate the body temperature,therefore surgeons had the discretion to decide for a higher target temperature and this was systematically applied determining a statistically different temperature during arrest in the 2 groups. In contrast, patients in the ACP group were maintained at a nasopharyngeal temperature of 24–26 °C and a rectal temperature of 26–28 °C in accordance with previous reports [26, 27]. The higher temperature in the RIVP + ACP group shortened CPB time, which may be benefit to shorten the ventilation time via attenuating the inflammatory response in the lung induced by the artificial material of the CPB circuit [28].

Interestingly, patients in the RIVP + ACP group also showed lower risk of TND than patients in the ACP group. Because of the limited influence of various cerebral perfusion strategies on the neurological outcome [29], the difference in TND is unlikely blamed of the different cannulation strategies in two groups. We speculate that at least three processes may contribute to the results. First, the warmer circulatory arrest temperature may help lower TND. At the same perfusion pressure of 50–60 mmHg, ACP provided higher cerebral perfusion flow (by 1.4 mL/kg/min) in patients undergoing RIVP + ACP than in those receiving ACP alone, suggesting that a 2.5 °C increase in body temperature may provide better vascular tone and allow more blood flow to the brain. Consistent with this hypothesis, the RIVP + ACP group showed a lower rate of bilateral ACP than the ACP group. Second, the collaterals between the inferior and superior vena cava might shunt the RIVP flow and compete with ACP, which may attribute to a more uniform distribution of cerebral perfusion. The possibility was confirmed in mice when it was shown that methylene blue appeared in the brain if it was perfused from inferior vena cava (without ACP) (data not shown). Third, the protection of RIVP on gastrointestinal tract from ischemic injury might also play a role in lower rate of TND via the gut-brain axis. This complex interaction network between the central nervous system and the peripheral intestinal functions, showed that the proper maintenance of gastrointestinal homeostasis would benefit the affect, motivation, and cognitive functions [30]. The possibility should be explored in the next studies.

Patients who underwent RIVP + ACP also showed lower blood transfusion requirements than those who underwent ACP alone. This may be because the RIVP + ACP group underwent rewarming an average around one hour shorter than ACP group. The ACP group required longer rewarming because of surgical bleeding, which necessitated additional stitches between the graft and descending aorta to ensure hemostasis. Probably in connection with their greater surgical bleeding, the ACP group showed higher chest drainage, and a higher proportion of patients required additional stitches after circulatory recovery.

Our pilot study has several limitations. First, the sample was small and involved patients at only one institution. This increases the risk that our study was underpowered, which may help explain the relatively small differences that we observed. In addition, the small size prevented us from completely eliminating bias due to inter-group differences in pre-operative variables. Even so, this pilot study has helped establish the feasibility and minimal sample size for the larger prospective trial. Second, patients undergoing RIVP + ACP had higher body temperatures during circulatory arrest and shorter CPB duration, so we could not isolate the effects of each of these factors from the effects of RIVP itself. Third, we took the pressure in the inferior vena cava to equal the circuit pressure immediately after the RIVP pump. It might overestimate the pressure in the patient, as a result of pressure drop along the cannula. Fourth, the outcomes in this study were limited to events in-hospital or 30 days after surgery, so we do not have information on the impact of RIVP on long-term outcomes.

## Conclusion

Our data suggest that in patients undergoing TARS did not show superiority of ACP-RIVP over ACP for the primary outcome. However, RIVP + ACP might be associated with lower incidence of postoperative transient neurologic deficits, shorter intubation and less blood products requirements than ACP alone. Multi-center, randomized trials with larger samples should be conducted to investigate the safety and efficacy of RIVP, especially since it can, in principle, be applied to all patients with type A acute aortic dissection who are scheduled for TARS, and would be broadly useful in any aortic surgery requiring lower body aortic occlusion.

## Supplementary Information

**Additional file 1**. Retrograde inferior vena caval perfusion (RIVP) with antegrade cerebral perfusion (ACP) is easy to be performed. It might be associated with better outcomes than ACP alone in patients undergoing total aortic arch replacement surgery

**Additional file 2**. Authorship form.

## Data Availability

Data sharing in the current study are available from the corresponding author on reasonable request.

## Abbreviations

AAAD: Acute type A aortic dissection
TARS: Total aortic arch replacement surgery
DHCA: Deep hypothermic circulatory arrest
ACP: Antegrade cerebral perfusion
RCP: Retrograde cerebral perfusion
MHCA: Moderate hypothermic circulatory arrest
RIVP: Retrograde inferior vena caval perfusion
CPB: Cardiopulmonary bypass
TND: Transient neurological deficits
ORs: Odds ratios
BMI: Body mass index

## References

[1] Isselbacher EM. Epidemiology of thoracic aortic aneurysms, aortic dissection, intramular hematoma, and penetrating atherosclerotic ulcers. In: Baliga RR, Nienaber CA, Isselbacher EM, Eagle KA, editors. Aortic dissection and related syndromes. New York: Springer Science 2007. p. 3e15.

[2] Tsai TT, Trimarchi S, Nienaber CA (2009). Acute aortic dissection: perspectives from the International registry of acute aorticdissection (IRAD). Eur J Vasc Endovasc Surg..

[3] Moon MR (2009). Approach to the treatment of aortic dissection. Surg Clin North Am..

[4] Griepp RB, Stinson EB, Hollingsworth JF, Buehler D (1975). Prosthetic replacement of the aortic arch. J Thorac Cardiovasc Surg.

[5] Safi HJ, Miller CC, Lee TY, Estrera AL (2011). Repair of ascending and transverse aortic arch. J Thorac Cardiovasc Surg..

[6] Ehrlich M, Fang WC, Grabenwoger M, Cartes-Zumelzu F, Wolner E, Havel M. Perioperative risk factors for mortality inpatients with acute type a aortic dissection. Circulation 1998;98(19) Suppl.: II294–8. 9852917

[7] Bavaria JE, Woo YJ, Hall RA, Wahl PM, Acker MA, Gardner TJ (1996). Circulatory management with retrograde cerebral perfusion for acute type A aortic dissection. Circulation.

[8] Augoustides JG, Floyd TF, McGarvey ML, Ochroch EA, Pochettino A, Fulford S (2005). Major clinical outcomes in adults undergoing thoracicaortic surgery requiring deep hypothermic circulatory arrest: quantification of organ-based perioperative outcome and detection of opportunities for perioperative intervention. J Cardiothorac Vasc Anesth..

[9] Arnaoutakis GJ, Bihorac A, Martin TD, Hess PJ, Klodell CT, Ejaz AA (2007). RIFLE criteria for acute kidney injury in aortic arch surgery. J Thorac Cardiovasc Surg.

[10] Leshnower BG, Myung RJ, Kilgo PD, Vassiliades TA, Vega JD, Thourani VH (2010). Moderate hypothermia and unilateral selective antegrade cerebral perfusion: a contemporary cerebral protection strategy for aortic arch surgery. Ann Thorac Surg.

[11] Bakhtiary F, Dogan S, Dzemali O, Kleine P, Moritz A, Aybek T (2006). Mild hypothermia (32°C) and antegrade cerebral perfusion in aortic arch operations. J Thorac Cardiovasc Surg..

[12] Hagl C, Ergin MA, Galla JD, Lansman SL, McCullough JN, Spielvogel D, Sfeir P, Bodian CA, Griepp RB (2001). Neurologic outcome after ascending aorta-aortic arch operations: effect of brain protection technique in high-risk patients. J Thorac Cardiov Surg..

[13] Shenkman Z, Elami A, Weiss YG, Glantz L, Milgalter E, Drenger B, Burrows FA, Shir Y (1997). Cerebral protection using retrograde cerebral perfusion during hypothermic circulatory arrest. Can J Anaesth..

[14] Usui A, Miyata H, Ueda Y, Motomura N, Takamoto S (2012). Risk-adjusted and casematched comparative study between antegrade and retrograde cerebral perfusion during aortic arch surgery: based on the Japan Adult Cardiovascular Surgery Database: the Japan Cardiovascular Surgery Database Organization. Gen Thorac Cardiovasc Surg..

[15] Qian H, Hu J, Du L, Xue Y, Meng W, Zhang EY (2013). Modified hypothermic circulatory arrest for emergent repair of acute aortic dissection type a: a single-center experience. J Cardiothorac Surg..

[16] Okita Y, Miyata H, Motomura N, Takamoto S, Japan Cardiovascular Surgery Database Organization. A study of brain protection during total arch replacement comparing antegrade cerebral perfusion versus hypothermic circulatory arrest, with or without retrograde cerebral perfusion: Analysis based on the Japan Adult Cardiovascular Surgery Database. J Thorac Cardiovasc Surg. 2015;149(2 Suppl):S65–73. doi:10.1016/j.jtcvs.2014.08.070. 10.1016/j.jtcvs.2014.08.070 25439767

[17] Apaydin AZ, Islamoglu F, Askar FZ, Engin C, Posacioglu H, Yagdi T, Ayik F (2009). Immediate clinical outcome after prolonged periods of brain protection: retrospective comparison of hypothermic circulatory arrest, retrograde, and antegrade perfusion. J Card Surg..

[18] Hiraoka A, Chikazawa G, Totsugawa T, Sakaguchi T, Tamura K, Yoshitaka H (2014). Acute kidney injury after total aortic arch repair with moderate hypothermic circulatory arrest. J Card Surg.

[19] Roh GU, Lee JW, Nam SB, Lee J, Choi JR, Shim YH (2012). Incidence and risk factors of acute kidney injury after thoracic aortic surgery for acute dissection. Ann Thorac Surg..

[20] Lin J, Xiong J, Luo M, Tan Z, Wu Z, Guo Y, Du L (2019). Ann Thorac Surg..

[21] Poon SS, Theologou T, Harrington D, Kuduvalli M, Oo A, Field M. Hemiarch versus total aortic arch replacement in acute type A dissection: a systematic review and meta-analysis. Ann Cardiothorac Surg . 2016;5(3):156–73. http:// doi: 10.21037/acs.2016.05.06. 10.21037/acs.2016.05.06 PMC4893527 27386403

[22] Lin J, Tan Z, Yao H, Hu XL, Zhang DF, Zhao Y, Xiong J, Dou B, Zhu X, Wu Z, Guo Y, Kang D, Du L. Retrograde Inferior Vena caval Perfusion for Total Aortic Arch Replacement Surgery (RIVP-TARS): study protocol for a multicenter, randomized controlled trial. 2019;20(1):232. doi: 10.1186/s13063-019-3319-2. 10.1186/s13063-019-3319-2 PMC6480889 31014386

[23] Ma WG, Zheng J, Dong SB, Lu W, Sun K, Qi RD, Liu YM, Zhu JM, Chang Q, Sun LZ (2013). Sun’s procedure of total arch replacement using a tetrafurcated graft with stented elephant trunk implantation: analysis of early outcome in 398 patients with acute type A aortic dissection. Ann Cardiothorac Surg.

[24] Andersson B, Andersson R, Brandt J, Höglund P, Algotsson L, Nilsson J (2010). Gastrointestinal complications after cardiac surgery - improved risk stratification using a new scoring model. Interact Cardiovasc Thorac Surg..

[25] Künzli A, Zingg PO, Zünd G, Leskosek B, von Segesser LK (2006). Does retrograde cerebral perfusion via superior vena cava cannulation protect the brain?. Eur J Cardiothorac Surg..

[26] Andersson B, Andersson R, Brandt J, Höglund P, Algotsson L, Nilsson J (2010). Gastrointestinal complications after cardiac surgery - improved risk stratification using a new scoring model. Interact Cardiovasc Thorac Surg..

[27] Künzli A, Zingg PO, Zünd G, Leskosek B, von Segesser LK. Does retrograde cerebral perfusion via superior vena cava cannulation protect the brain? 2006;30(6):906–9. doi:10.1016/j.ejcts.2006.08.024. 10.1016/j.ejcts.2006.08.024 17071098

[28] Day JRS, Taylor KM (2005). The systemic inflammatory response syndrome and cardiopulmonary bypass. Int J Surg.

[29] Angeloni E, Benedetto U, Takkenberg JJ, Stigliano I, Roscitano A, Melina G, Sinatra R (2014). Unilateral versus bilateral antegrade cerebral protection during circulatory arrest in aortic surgery: a meta-analysis of 5100 patients. J Thorac Cardiovasc Surg..

[30] Carabotti M, Scirocco A, Maselli MA, Severi C (2015). The gut-brain axis: interactions between enteric microbiota, central and enteric nervous systems. Ann Gastroenterol.

