# Supplementary material for: Retrograde inferior vena caval perfusion for total aortic arch replacement surgery: a randomized pilot study
Source: BMC Cardiovasc Disord. 2021 Apr 20;21:193. doi: 10.1186/s12872-021-02002-9 (PMC8056667; doi:10.1186/s12872-021-02002-9)
Supplement: Supplementary file 2 — Additional file 2. Authorship form. [file 12872_2021_2002_MOESM2_ESM.docx]

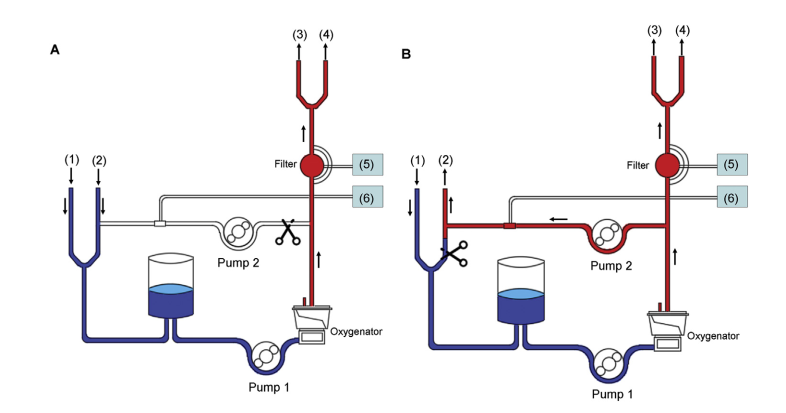
**Schematic of the bypass circuit and its connection with the body.** (A) Before and after retrograde inferior vena caval perfusion, venous blood from the (1) superior vena cava and (2) inferior vena cava is returned to the reservoir and driven by pump 1 to the oxygenator and then back to the artery (3) via a filter, where pressure is monitored (5). (B) During retrograde inferior vena caval perfusion, venous blood from the (1) superior vena cava is returned to the reservoir and driven by pump 1 to the oxygenator. Part of the oxygenated blood is routed for antegrade cerebral perfusion (4), while the other part is driven by pump 2 back to the lower body via the (2) inferior vena cava. Circulatory pressure is monitored (6). See also *Ann Thorac Surg* 2019;107:e67–9.

**Supplementary definitions**

1. The surgical period was defined as the interval from skin incision to the last stitch, which was recorded on the anesthesia chart.
2. Stroke was defined as a postoperative stroke (i.e., any confirmed neurological deficit of abrupt onset caused by a disturbance in blood supply to the brain) that did not resolve within 24 hours [1].
3. Temporary neurologic deficit (TND) was regarded as the occurrence of postoperative agitation, confusion, delirium, obtundation or a transient focal neurologic deficit (resolution within 72 hours) without any evidence of new structural abnormality on computed tomography or magnetic resonance [2].

| **Score** | **Motor function** | **Deficit** |
| --- | --- | --- |
| 0 | No lower extremity movement | Paraplegia |
| 1 | Lower extremity movement without gravity | Paraplegia |
| 2 | Lower extremity motion against gravity | Paraplegia |
| 3 | Able to stand with assistance | Paraparesis |
| 4 | Able to walk with assistance | Paraparesis |
| 5 | Normal | Normal |

1. Paraparesis was defined as a Tarlov score 3–4 [3].
2. Renal failure was defined when patients were given renal replacement therapy (RRT) because they were classified as grade F according to the RIFLE criteria: (a) serum creatinine ≥ 4 mg/dL or an increase of at least three times the baseline level, or > 75% decline in the estimated glomerular filtration rate; and/or urinary output < 0.3 mL/kg/h for 24 h or anuria for 12 h. We defined RRT as intermittent hemodialysis or continuous venovenous hemofiltration [4].

**References**

1. The Society of Thoracic Surgery National Database specifications (http://www.sts. org)
2. Misfeld M, Leontyev S, Borger MA, Gindensperger O, Lehmann S, Legare JF, Mohr FW: What is the best strategy for brain protection in patients undergoing aortic arch surgery? A single center experience of 636 patients. Ann Thorac Surg 2012, 93:1502–1508.
3. Bisdas T, Panuccio G, Sugimoto M, Torsello G, Austermann M. Risk factors for spinal cord  ischemia after endovascular repair of thoracoabdominal aortic aneurysms. J Vasc Surg. 2015;61:1408–16.
4. Bellomo R, Ronco C, Kellum J, Mehta RL, Palevsky P, Acute Dialysis Quality Initiative Workgroup. Acute renal failure—definition, outcome measures, animal models, fluid therapy and information technology needs: the Second International Consensus Conference of the Acute Dialysis Quality Initiative (ADQI) Group. Crit Care. 2004;8:R204.

Supplementary table:

**Table S1. RIFLE classification of patients treated with ACP or RIVP +ACP during TARS, n (%)**

|  | **ACP group**  **(n=38)** | **RIVP+ACP group**  **(n=38)** | ***P value*** |
| --- | --- | --- | --- |
| **RIFLE classification** | |  | 0.28 |
| 0 | 9 (23.7) | 14 (36.8) |  |
| Ⅰ | 12 (31.6) | 11 (28.9) |  |
| II | 10 (26.3) | 7 (18.4) |  |
| III | 7 (18.4) | 6 (15.8) |  |

ACP, antegrade cerebral perfusion; RIVP, retrograde inferior vena caval perfusion; TARS, total aortic replacement surgery

**Table S2. Chest drainage and hemoglobin levels in ACP or RIVP +ACP groups after TARS.**

| **Time point** | **ACP group**  **(n=38)** | **RIVP+ACP group**  **(n=38)** | ***P value*** |
| --- | --- | --- | --- |
| **Chest drainage (mL)** |  |  | 0.001 |
| 4 h after surgery | 190 (100,333) | 110 (63,150) |  |
| 12 h after surgery | 403 (303,740) | 225 (153,390) |  |
| 24 h after surgery | 615 (438,941) | 395 (308,610) |  |
| 48 h after surgery | 966 (755,1410) | 623 (493,961) |  |
| 72 h after surgery | 1177 (1000,1780) | 950 (655,1330) |  |
| **Hemoglobin (g/L)** | |  | 0.083 |
| ICU arrival | 103 (93,112) | 101 (94,111) |  |
| 4 h after surgery | 92 (81,100) | 93 (85,105) |  |
| 12 h after surgery | 92 (87,101) | 95 (86,106) |  |
| 24 h after surgery | 89 (83,98) | 94 (89,99) |  |
| 48 h after surgery | 90 (83,95) | 96 (89,100) |  |
| 72 h after surgery | 88 (84,94) | 95 (91,98) |  |

Values are median (interquartile range)

ACP, antegrade cerebral perfusion; RIVP, retrograde inferior vena caval perfusion; TARS, total aortic replacement surgery
